# Dihydroartemisinin Inhibits Laser-Induced Choroidal Neovascularization in a Mouse Model of Neovascular AMD

**DOI:** 10.3389/fphar.2022.838263

**Published:** 2022-02-18

**Authors:** Xun Li, Sheng Gao, Yun Zhang, Mei Xin, Cheng Zuo, Naihong Yan, Qingjie Xia, Meixia Zhang

**Affiliations:** ^1^ Department of Ophthalmology, West China Hospital, Sichuan University, Chengdu, China; ^2^ Research Laboratory of Ophthalmology and Vision Science, West China Hospital, Sichuan University, Chengdu, China; ^3^ Research Laboratory of Macular Disease, West China Hospital, Sichuan University, Chengdu, China; ^4^ Department of Ophthalmology, Chengdu First People’s Hospital, Chengdu, China; ^5^ Department of Ophthalmology, The Third People’s Hospital of Chengdu, Chengdu, China; ^6^ Laboratory of Neurological Disease, Translational Neuroscience Center, West China Hospital, Sichuan University, Chengdu, China

**Keywords:** dihydroartemisinin (DHA), neovascular age-related macular degeneration (nAMD), choroidal neovascularization, NF-κB signaling pathway, antiangiogenic efficacy

## Abstract

**Purpose:** Choroidal neovascularization (CNV) is the main pathogenic process and a leading cause of severe vision loss in neovascular age-related macular degeneration (AMD). We investigated the antiangiogenic efficacy of dihydroartemisinin (DHA) in an experimental laser-induced CNV mouse model.

**Methods:** After fluorescein angiography confirmed that CNV was induced by laser photocoagulation in C57BL/6J mice, DHA or vehicle was given by intragastric administration once a day. On day 6 and day 12, fluorescein angiography, optic coherence tomography, and flat-mounting analysis were performed to grade CNV leakage, measure CNV thickness and evaluate CNV areas, respectively. Immunofluorescence staining and Western blot analysis were performed to evaluate the expression of NF-κB, VEGF, and VEGFR2. To confirm the safety of intragastric DHA application, changes in retinal morphology and neural cell apoptosis were tested by histopathological examination and TUNEL assay, and retinal function was determined by electroretinogram (ERG).

**Results:** Intragastric administration of DHA significantly suppressed CNV leakage and CNV formation in both thickness and area. Immunofluorescence showed that DHA suppressed VEGFR2 and NF-κB p65 expression in laser-induced lesions. Compared to the normal group, the protein expression of VEGF, VGFER2, NF-κB p65, and NF-κB1 p50 increased significantly in the vehicle group after laser photocoagulation, while it was profoundly inhibited by DHA treatment. In addition, histopathological examination, TUNEL analysis, and ERG test showed no obvious evidence of retinal toxicity caused by DHA.

**Conclusion:** Systemic administration of DHA can effectively inhibit laser-induced CNV formation in mice, which might be due to the suppression of the classic NF-κB signaling pathway and downregulation of VEGFR2 and VEGF expression. The current results suggest that DHA could be a natural potential alternative therapeutic strategy for neovascular AMD.

## Introduction

Age-related macular degeneration (AMD) has been the most common cause of permanent visual damage and public health problems due to the aging population in the developed world (Chakravarthy and Peto, 2020; Apte, 2021). Choroidal neovascularization (CNV) is the main pathogenic process and a leading cause of severe vision loss in neovascular AMD (Jaffe et al., 2019; Zhao et al., 2019). Since the increased expression of vascular endothelial growth factor (VEGF) is crucial during pathogenic angiogenesis, anti-VEGF therapies have been successfully used to treat neovascular AMD (Wong et al., 2016). However, these treatments have several limitations, such as the requirement of repeat intravitreal injections, the development of tolerance, and the heavy burden on patients caused by the high costs. Currently, there are no satisfactory noninvasive treatments for neovascular AMD (Mehta et al., 2018). Thus, there is an urgency to seek cost-effective, less invasive, and more durable alternative therapies for CNV in AMD patients.

Dihydroartemisinin (DHA), an artificial semisynthetic derivative of artemisinin and the main metabolite of artemisinin in the body, has characteristics of high activity and low toxicity. In addition to its excellent antimalarial effect, the antiangiogenic efficacy of DHA has been shown in several cancer studies, including myeloma, leukemia, ovarian cancer, Lewis lung cancer and pancreatic cancer (Efferth, 2017). *In vitro*, DHA inhibits the growth, proliferation, migration and tube formation of endothelial cells by downregulating VEGF, all of which are closely linked with angiogenesis (Guo et al., 2014). Moreover, DHA regulates VEGFR2 promoter activity through the p65 binding motif and decreases the binding activity of p65 and the VEGFR2 promoter, suggesting that inhibition of the NF-κB pathway plays a major role in mediating the antiangiogenic effects of DHA (Dong et al., 2014). As a prototypical proinflammatory signaling pathway, recent studies have shown that NF-κB regulates many angiogenic factors in the development of cancers (Karin, 2006). Moreover, NF-κB was detected in the retinal pigment epithelium of human eyes with advanced stages of AMD (Feng et al., 2017). The inhibition of NF-κB by genetic deletion or pharmacological inhibition of IKK2 significantly reduces laser-induced CNV, suggesting a key role of NF-κB in CNV formation (Gaddipati et al., 2015; Ghosh et al., 2017). However, whether DHA attenuates choroidal neovascularization *in vivo* remains unknown.

In this study, we investigated the effect of DHA on laser-induced CNV in a mouse model to explore its potential application as a new therapy for neovascular AMD and preliminarily explored the underlying mechanism.

## Materials and Methods

### Animals and Anesthesia

Male C57BL/6J mice (DOSSY EXPERIMENTAL ANIMALS CO. LTD., Chengdu, China) aged 6–8 weeks old and weighing 18–20 g were used to minimize variability in the study. Animals were housed in metal breeding cages with free access to food and water in a room with a 12-h/12-h light/dark cycle. The humidity and temperature were maintained at 60% and 24–26°C, respectively. All animal procedures were approved by the Animal Care and Use Committee of Sichuan University and adhered to the Association for Research in Vision and Ophthalmology (ARVO) Statement for the Use of Animals in Ophthalmic and Vision Research. During all procedures, the mice were anesthetized with an intraperitoneal injection of 2% pentobarbital sodium (45 mg/kg body weight), and pupils were dilated with topical 5% tropicamide (Santen Pharmaceutical Co., LTD., Osaka, Japan). At different time points after laser-induced photocoagulation, the mice were euthanized by cervical dislocation.

### Establishment of Mouse Choroidal Neovascularization Model

After dilating pupils, mice were placed on a platform under a slit lamp, and the corneas were anesthetized with oxybuprocaine hydrochloride eye drops (Santen Pharmaceutical Co., LTD., Osaka, Japan). 532 nm argon laser-induced photocoagulation was used to disrupt Bruch’s membrane bilaterally in each mouse. Four spots of laser photocoagulation in the posterior pole of the retina were created with a power of 150–200 mW, duration of 100 m s, and 50 μm spot size using a slit lamp delivery system with a handheld coverslip as a contact lens. The laser spots are located approximately two to three optic disc diameters away from the optic nerve head, avoiding the main vessels. The appearance of a white bubble, which indicates a break of Bruch’s membrane, is an important factor to obtain CNV, and only burns causing bubble formation were included in subsequent experiments. Spots with hemorrhage or failing to develop a bubble were excluded from the analysis.

### Administration of DHA and Control Vehicle

**FIGURE 1:**
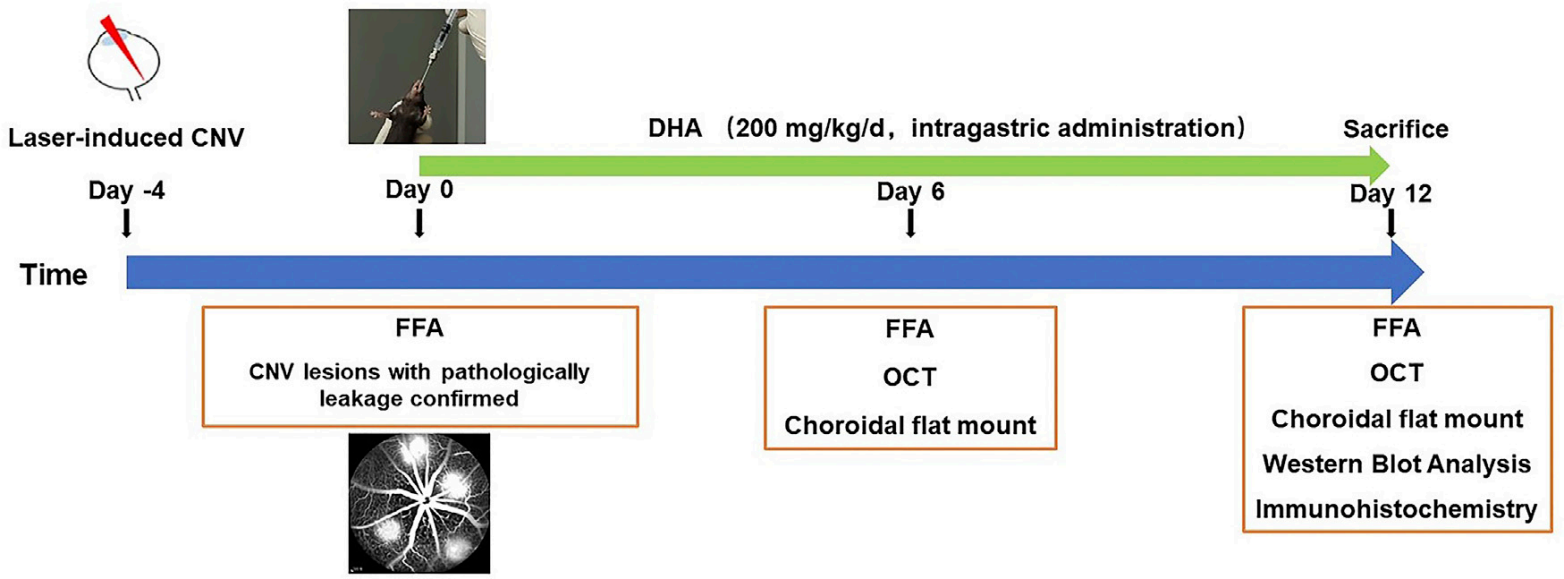
Experimental schedules to evaluate the effect of DHA on laser-induced choroidal neovascularization CNV in a mouse model. After fluorescein angiography confirmed that CNV was induced, DHA or vehicle was intragastrically administered once a day for 12 continuous days. CNV, Choroidal Neovascularization; DHA: Dihydroartemisinin; FFA: Fundus Fluorescein Angiography; OCT: Optic Coherence Tomography.

DHA was supplied by the Center for Translational Neuroscience, West China Hospital, Sichuan University, and it was dissolved in 10% dimethyl sulfoxide (DMSO). Animals were randomly divided into three groups: control (vehicle only), DHA, and ranibizumab. Four days after laser photocoagulation, the DHA group received intragastric administration of DHA at a dose of 200 mg/kg/d for 12 consecutive days, and the control group mice received the same volume of vehicle (Figure 1). The ranibizumab group mice received an intravitreal injection of 2 μL (20 μg) ranibizumab as a positive control. Mice were anesthetized and pupils were dilated with topical 5% tropicamide. A shelving puncture of the sclera was made 1 mm behind the limbus with a 32-gauge needle, then a 33-gauge needle (Hamilton Bonaduz AG, Bonaduz, Switzerland) was inserted with a 45° injection angle into the vitreous. The direction and location of the needle was monitored through the microscope. The overall health of each mouse was evaluated by assessing its weight, general appearance, and alertness. All mice remained alert and responsive, and there were no adverse effects during the study period.

### Fundus Fluorescein Angiography

Fundus fluorescein angiography (FFA) was performed on anesthetized mice using a Spectralis HRA + OCT ophthalmic diagnostic apparatus (Heidelberg, Germany) at the time points. Early-phase and late-phase FFA images were recorded at 1 to 2 min and after 5 min, respectively, after intraperitoneal injection of 0.2 ml 2% fluorescein sodium solution (Baiyunshan Mingxing Pharmaceutical Co., Ltd., Guangzhou, China). Images were evaluated by two masked retinal specialists who were not involved in laser photocoagulation or angiography. Then, lesions were graded according to a previously published grading system: 0, “no leakage,” faint hyperfluorescence or mottled fluorescence; 1, “questionable leakage,” hyperfluorescence lesion without a progressive increase in size or intensity; 2, “leaky,” hyperfluorescence increasing in intensity but not in size; and 3, “pathologically significant leakage,” hyperfluorescence increasing both in size and intensity (Ozone et al., 2016). Four days after laser photocoagulation, we performed FFA analysis and chose lesions with pathologically significant leakage for further observation because only these lesions had the clinical meaning of CNV. Then, the DHA was administered, as shown in Figure 1. Late-phase images were chosen for analysis of fluorescence leakage and leakage areas, which corresponded with the clearance time of fluorescence from the vasculature and to avoid interference with the observation of hyperfluorescence from CNV.

### Spectral Domain-Optic Coherence Tomography (SD-OCT)

SD-OCT was performed using a Heidelberg Spectralis HRA + OCT (Heidelberg, Germany). The cornea was covered with hyaluronan gel (Freda, China) before the examination. For each CNV lesion, SD-OCT high-resolution multilinear horizontal scanning images were taken to calculate the thickness of the CNV lesion, which was defined as a spindle-shaped hyperreflective area at the subretinal space. Only the scan that passed through the center of the lesion was chosen to evaluate the CNV central thickness (CCT) (Zhao et al., 2018).

### Flat-Mount Staining and CNV Size Measurement

Mice were euthanized, and eyes were enucleated and fixed in 4% paraformaldehyde solution for approximately 30 min. After washing in PBS, the anterior of the eye was cut off, and the whole retinas were removed carefully from the eyecups. To flatten the eyecups, four radial incisions in the remaining RPE-choroid-sclera complexes were made from the edge to the optic nerve head with the RPE facing up. Then, the complexes were washed in tris-buffered saline (0.5% BSA, 0.2% Tween-20, and 0.1% Triton X-100), incubated with isolectin B4 (1:200, Sigma–Aldrich, St Louis, MO, United States) overnight at 4°C, and mounted onto glass slides. The images were captured with a fluorescence microscope (Carl Zeiss, Germany), and the CNV lesion area was measured using ImageJ software after normalizing for different background fluorescence (NIH, Bethesda, MD, United States).

### Immunohistochemistry

The eyecups were embedded in an optimal cutting temperature compound (Tissue Tek; Miles Laboratories, Elkhart, IN, United States) and snap-frozen. 10-μm thick cryostat sections were cut at-20°C, air dried, and incubated in blocking solution (5% normal donkey serum, 1% BSA, and 0.03% Triton X-100 in 1× PBS) for 1 hour at room temperature. The sections were stained with monoclonal antibodies against NF-κB p65 (CST, 8,242, Beverly, MA, United States) or with polyclonal antibodies against VEGFR2 (CST, 2,478, Beverly, MA, United States) at 4 °C overnight and then incubated with corresponding secondary antibodies conjugated to Alexa Fluor 488 (Invitrogen, United States) for 1 h at room temperature in the dark, while the nuclei were stained with 4′6-diamidino-2-phenylindole (DAPI; Sigma Aldrich). A fluorescence microscope (Carl Zeiss, Germany) was used to take pictures.

### Western Blot Analysis of NF-κB Signaling, VEGF, and VEGFR2

To determine whether DHA treatment affected the NF-κB signaling pathway, RPE-choroid complexes were microsurgically isolated and transformed immediately into 200 μL RIPA buffer (Beyotime Institute of Biotechnology, Haimen, China) supplemented with 1% protease inhibitor cocktail (Beyotime) at 4 °C. After electric homogenization, lysates were placed on ice for 60 min and centrifuged at 12,000 rpm for 20 min at 4°C. Samples were collected and preserved at-80°C. Protein concentrations were measured by the BCA Protein Assay Kit (Beyotime) with bovine serum albumin as the standard. Each sample containing 20 μg of total protein was separated by sodium dodecyl sulfate–polyacrylamide gel electrophoresis (SDS–PAGE) and then transferred to a polyvinylidene fluoride (PVDF) membrane (Millipore, Billerica, MA, United States). After blocking with 5% skim milk for 2 h at room temperature, the membranes were incubated with primary antibodies against NF-κB p65 (CST, 8,242, Beverly, MA, United States), VEGF (Abcam, 46,154, Cambridge, MA), VEGFR2 (CST, 2,478), NF-κB1 p105/p50 (CST, 13,586), NF-κB2 p100/p52 (CST, 37,359), Rel B (CST, 10,544) and *β*-actin (Beyotime, AA128) at 4 °C overnight. NF-κB1 p105/p50 Rabbit mAb was able to recognize endogenous levels of both the p105 precursor and p50 active form of NF-κB protein, as the same with NF-κB2 p100/p52 Rabbit mAb. The active forms of p50 and p52 were analyzed in this study to reflect the intracellular impact of DHA. The membranes were then incubated with goat anti-rabbit IgG (HRP)-conjugated or goat anti-mouse IgG (HRP)-conjugated antibodies for 2 h at room temperature.

### Electroretinogram (ERG), Histopathologic Analysis, and TUNEL Assay

Mice received intragastric administration of either DHA or vehicle once a day for three consecutive weeks. The overall health of the mice was evaluated by assessing their weight, breathing, alertness, and general appearance. The dark-adapted ERG (Diagnosys LLC) was recorded after DHA treatment. Mice were dark-adapted overnight and anesthetized with pentobarbital sodium, and pupils were dilated with topical 5% tropicamide. Full-field ERG was performed after inserting a ground electrode near the tail and a reference electrode on the back subcutaneously. A golden-ring electrode was gently positioned on the cornea. All procedures were performed under dim red light. Responses to brief flashes were analyzed primarily by measuring the amplitudes of the a- and b-waves. The amplitude of the a-wave was measured from the baseline to the maximum a-wave peak, and the b-wave was measured from the nadir of the a-wave to the apex of the b-wave peak. Then, the eyes were enucleated and fixed in 4% paraformaldehyde solution, conventionally dehydrated, embedded in paraffin wax and cut into 5.0 μm slices. As retinal thickness varies throughout the retina, all sections were parallel to the sagittal plane and through the optic nerve heads, and the regions were quantified at a constant distance from the optic nerve head. Sections were stained with hematoxylin-eosin (HE), observed, and photographed using a light microscope. Frozen sections of the eyeball were labeled by TdT-dUTP terminal nick-end labeling (TUNEL) with an apoptosis detection kit (Promega, United States) according to the manufacturer’s instructions. Nonspecific signals were detected by the omission of the enzyme reaction. The mouse retina at 24 h after ischemia (IOP raised to 110 mmHg for 1 h) and reperfusion was selected as a positive control group (RIR).

### Statistical Analysis

The data are expressed as the mean ± SEM where applicable. Statistical evaluation was conducted by one-way ANOVA followed by Tukey’s multiple comparisons test depending on the normality of the data. A value of *p* < .05 was considered statistically significant. Analysis was performed using the statistical software SPSS 26.0 (SPSS, Inc. Chicago, IL, United States).

## Results

### Antiangiogenic Effect of DHA on CNV

FFA was performed to evaluate the *in vivo* effect of DHA on CNV leakage. It was confirmed that angiographic leakage of CNV was less severe in the DHA group and ranibizumab group. The proportion of CNV formation with fluorescence leakage in each group is shown in Table 1. Pathologically significant leakage (grade 3 lesions) was observed in 26% of CNV lesions in the DHA group and 17% of CNV lesions in the ranibizumab group on day 6, both of which were less than that in the vehicle group (68%). This trend lasted until day 12. The average grade score was significantly lower in DHA (1.96 ± 0.77) or ranibizumab (1.74 ± 0.86) treated eyes compared with vehicle-treated eyes (2.58 ± 0.69) on day 6. And the similar situation was observed on day 12.

**TABLE 1:** CNV leakage grading.

	Vehicle	DHA	Ranibizumab	Vehicle	DHA	Ranibizumab
		Day 6			Day 12	
Number of CNV (grade 3) (Percentage)	13/19 (68%)	6/23 (26%)	4/23 (17%)	13/25 (52%)	5/24 (20.8%)	2/23 (9%)
Average Leakage Grade score	2.58 ± 0.69	1.96 ± 0.77^*^	1.74 ± 0.86^*^	2.32 ± 0.80	1.67 ± 0.82^#^	1.17 ± 0.78^#^

* *p*< 0.05 vs vehicle on day 6.

# *p*< 0.05 vs vehicle on day 12 (one-way ANOVA, followed by Tukey’s test).

### Inhibitory Effect of DHA on Laser-Induced CNV

**FIGURE 2:**
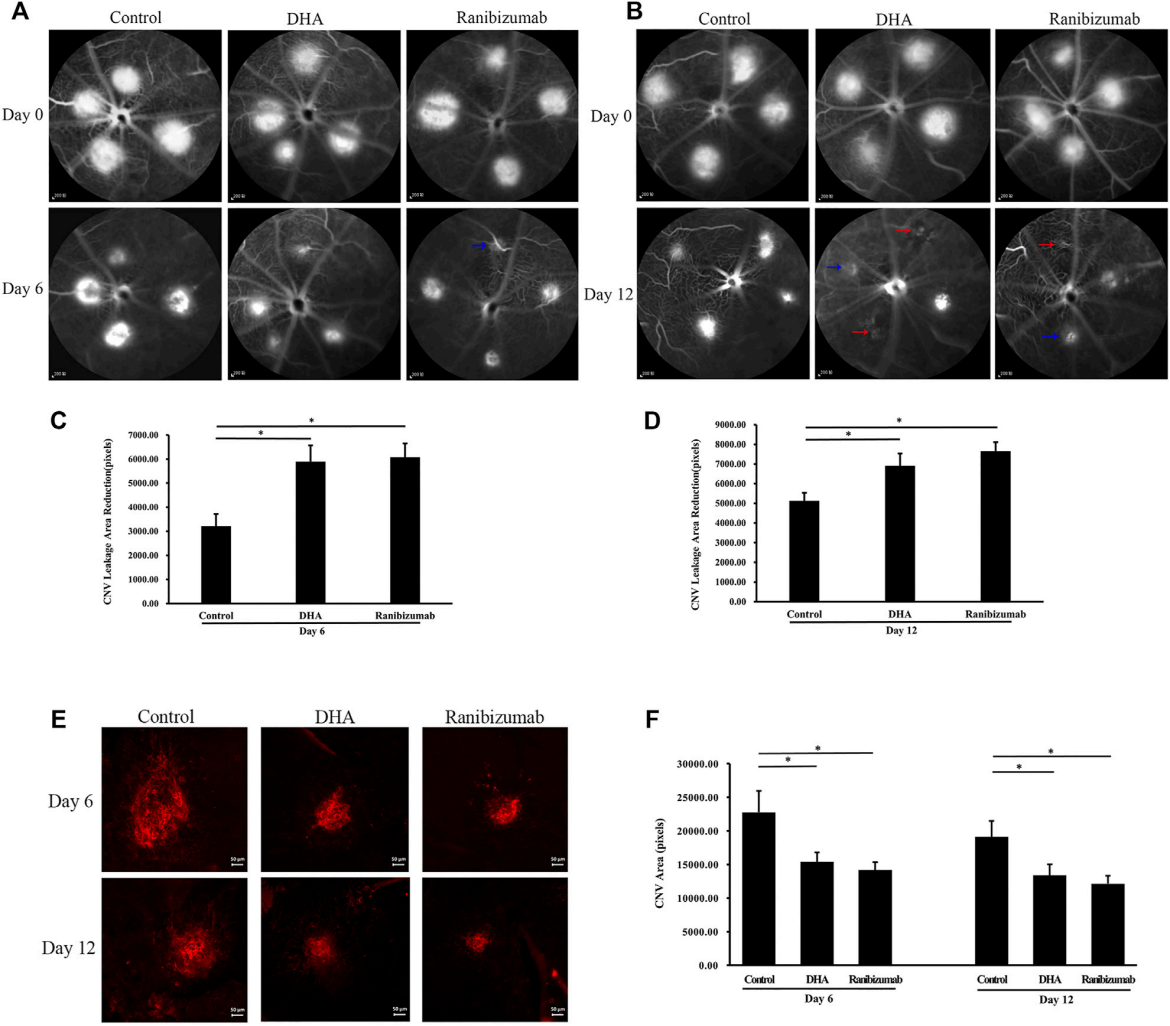
The effect of DHA on the CNV lesion area was shown by FFA and choroidal flat-mount staining. The CNV lesions were graded according to the intensity of the leaking fluorescence. Grade 1 was indicated by red arrows. Grade 2 was indicated by blue arrows. **(A,B)** Representative late-phase fluorescein angiograms on day 6 and day 12. Scale bar, 200 μm. **(C,D)** The reduction in the same CNV leakage area of the DHA group or ranibizumab group was significantly larger than that of the vehicle group on day 6 and day 12. **(E)** Representative choroidal flat-mount images of laser-induced vascular lesions (stained with isolectin B4) on day 6 and day 12. Scale bar, 50 μm. **(F)** The CNV area decreased significantly after DHA or ranibizumab treatment compared to that in the vehicle group. Data were analyzed by one-way ANOVA followed by Tukey’s multiple comparisons test. *p < .05. CNV: Choroidal Neovascularization; DHA, Dihydroartemisinin; FFA, Fundus Fluorescein Angiography.

The effect of DHA on the CNV lesion area was examined by FFA and choroidal flat-mount images, suggesting that the CNV area was significantly reduced after DHA treatment. Because of the advantage of FFA for continuously observing CNV lesions *in vivo*, the reduced leakage area of the same CNV was calculated (Figure 2). The mean reduction areas in the DHA group (5,895.84 ± 666.16 pixels, *p* < .05) and ranibizumab group (6,075.35 ± 576.87 pixels, *p* < .05) were larger than that in the vehicle group (3,202.80 ± 516.82 pixels) on day 6 (Figure 2C). After 12 days of administration, the mean reduction in the CNV leakage area was 6,901.25 ± 630.76 pixels in the DHA group and 7,657.69 ± 444.20 pixels in the ranibizumab group, both of which were significantly larger than that in the vehicle group (5,127.05 ± 407.49 pixels, *p* < .05) (Figure 2D).

To demonstrate the effect of DHA on the CNV area, the results of choroidal flat mounts also showed a distinct reduction in the CNV lesion area after DHA treatment (Figures 2E,F). On day 6, the CNV area decreased significantly in the DHA group (15,395.26 ± 1,372.16 pixels, *p* < 0.05) and the ranibizumab group (14,190.14 ± 1,160.56 pixels, *p* < .05) compared to the vehicle group (22,758 ± 3,216.07 pixels). The trend among these groups was similar on day 12.

**FIGURE 3:**
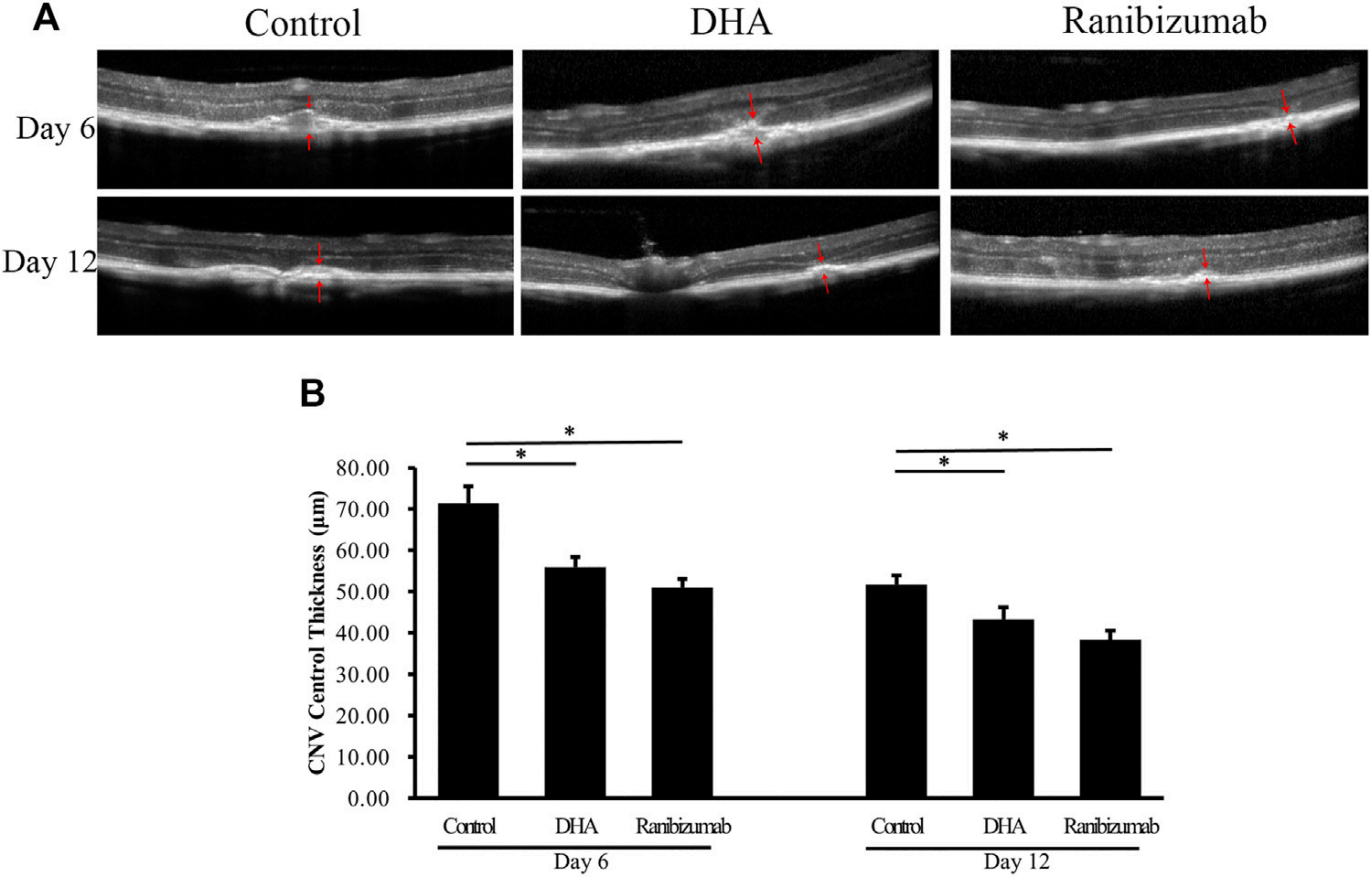
The effect of DHA on CNV central thickness (CCT) examined by SD-OCT images. The CCT of the spindle-shaped CNV lesion is outlined by the red arrows. **(A)** Representative SD-OCT images of CNV in mice administered vehicle, DHA, or ranibizumab. Scale bar, 50 μm. **(B)** Compared to that in the vehicle group, the CCT decreased significantly with DHA or ranibizumab treatment. Data were analyzed by one-way ANOVA followed by Tukey’s multiple comparisons test. *p < .05. CNV: Choroidal Neovascularization; DHA: Dihydroartemisinin.

The effect of DHA on CNV central thickness (CCT) was examined by SD-OCT analysis, showing that CCT decreased significantly after DHA treatment (Figure 3). The mean CCT in the DHA group was 55.96 ± 2.35 μm on day 6 and 43.30 ± 2.95 μm on day 12, and both were reduced markedly compared to the vehicle group on day 6 (71.42 ± 4.02 μm, *p* < .05) and day 12 (51.77 ± 2.14 μm, *p* < .05). In addition, CCT in eyes treated with ranibizumab was significantly lower than that in the vehicle group (*p* < .05), while there was no difference between the DHA group and the ranibizumab group (*p* > .05).

### Immunohistochemistry

**FIGURE 4:**
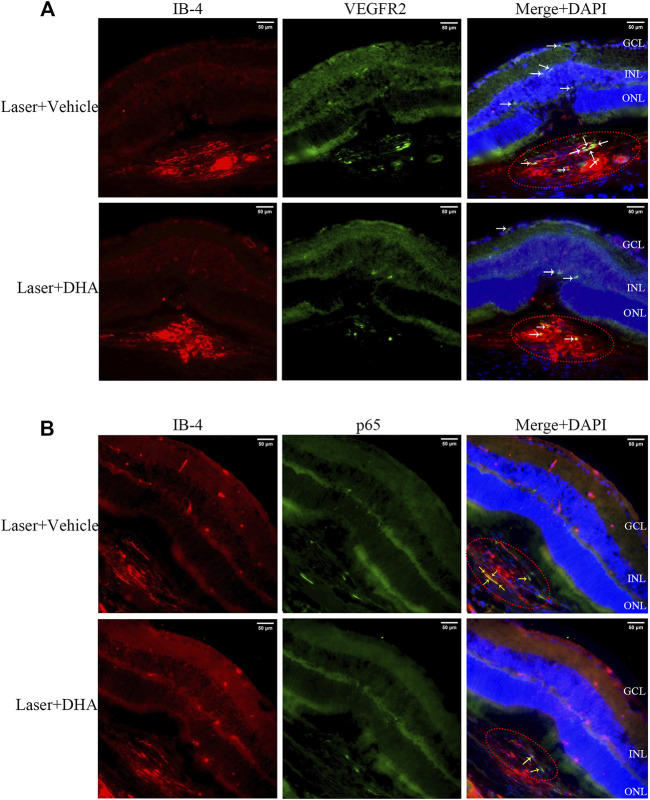
VEGFR2 and NF-κB p65 expression at the site of choroidal neovascularization (CNV) lesions analyzed by immunostaining images. The CNV lesion was indicated by the red ellipse. **(A)** The sections were fluorescently labeled with VEGFR2 antibody (green) and isolectin B4 (red). VEGFR2 had a little positive staining in retinal capillary endothelial cells in both the vehicle and dihydroartemisinin (DHA) groups. However, in CNV formation spots, the positive staining area of VEGFR2 in the DHA group was smaller than that in the vehicle group (white arrow). **(B)** The sections were fluorescently labeled with NF-κB p65 antibody (green) and isolectin B4 (red). The positive staining area of NF-κB p65 in the DHA group was significantly reduced compared to that in the vehicle group, which was mainly seen in CNV areas (yellow arrow). GCL, Ganglion Cell Layer; INL, Inner Nuclear Layer; ONL, Outer Nuclear Layer. Scale bar, 50 μm.

Immunostaining showed that intragastric administration of DHA reduced VEGFR2 and NF-κB p65 expression at the site of CNV formation (Figure 4). Whether in the vehicle group or the DHA group, VEGFR2 had a little positive staining in retinal capillary endothelial cells, and there was no significant difference. However, for CNV formation spots, the positive staining area of VEGFR2 in the DHA group was smaller than that in the vehicle-treated group (white arrow). Similarly, the positive staining of NF-κB p65 in CNV lesions in the DHA group was reduced compared to that in the vehicle group (yellow arrow).

### Inhibition of NF-κB Activation *in vivo* by Treatment With DHA

**FIGURE 5:**
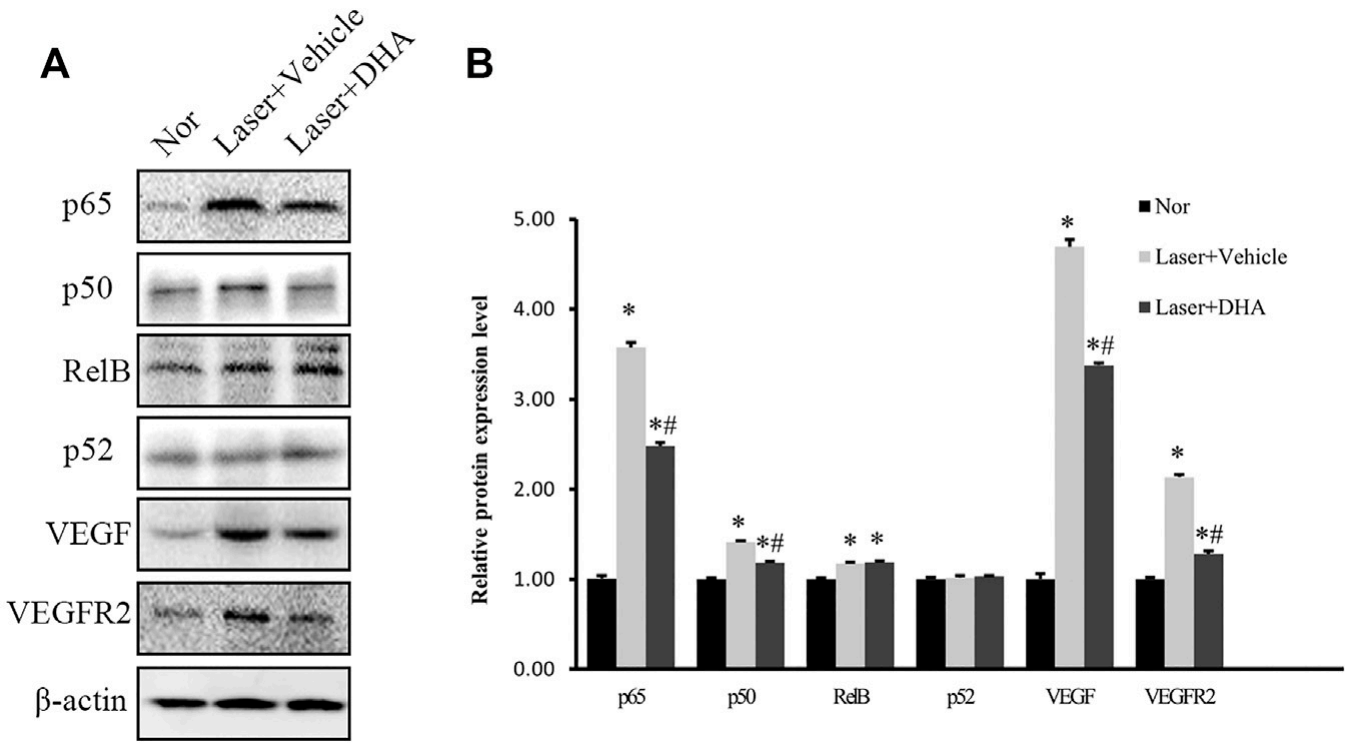
The impact of DHA on the protein expression levels of the NF-κB family, VEGF, and VEGFR2 in the RPE-choroid complex of a laser-induced CNV mouse model. Mice were intragastrically administered vehicle or DHA once a day for 12 days. **(A)** The typical Western blot results showed changes in NF-κB family, VEGF, and VEGFR2 protein expression. **(B)** The quantitative analysis results demonstrated that the expression levels of NF-κB p65, NF-κB1 p50, Rel B, VEGF, and VEGFR2 were markedly increased in the vehicle group compared to the normal control group. DHA significantly inhibited the expression of NF-κB p65, NF-κB1 p50, VEGF, and VEGFR2. *n* = 3 experiments; *p < .05 vs Nor; #*p* < .05 vs Laser + Vehicle; one-way ANOVA followed by Tukey’s test. CNV: Choroidal Neovascularization; DHA: Dihydroartemisinin.

To define the signaling pathway involved in the treatment of DHA, we focused on NF-κB as an upstream transcription factor of inflammatory mediators and analyzed the protein expression levels of NF-κB family members, such as NF-κB p65, NF-κB1 p105/p50, NF-κB2 p100/p52, and Rel B, as well as VEGF and VEGFR2 in homogenized RPE-choroid complex tissue. Figure 5 shows that the expression of NF-κB p65, NF-κB1/p50, Rel B, VEGF, and VEGFR2 was markedly increased in the vehicle-treated group compared to the normal control (*p* < .05). These effects were partially reversed by treatment with DHA. After DHA intervention, the protein levels of NF-κB p65, NF-κB1 p50, VEGF, and VEGFR2 were reduced significantly (*p* < .05), while the expression of Rel B did not decrease compared to the vehicle group (*p* > .05). However, the three groups did not differ significantly in terms of NF-κB2 p52 expression level (*p* > .05). Therefore, DHA inhibited the expression of NF-κB p65, NF-κB1 p50, VEGF, and VEGFR2 in eyes with laser-induced CNV.

### Toxicity of DHA on the Mouse Retina

**FIGURE 6:**
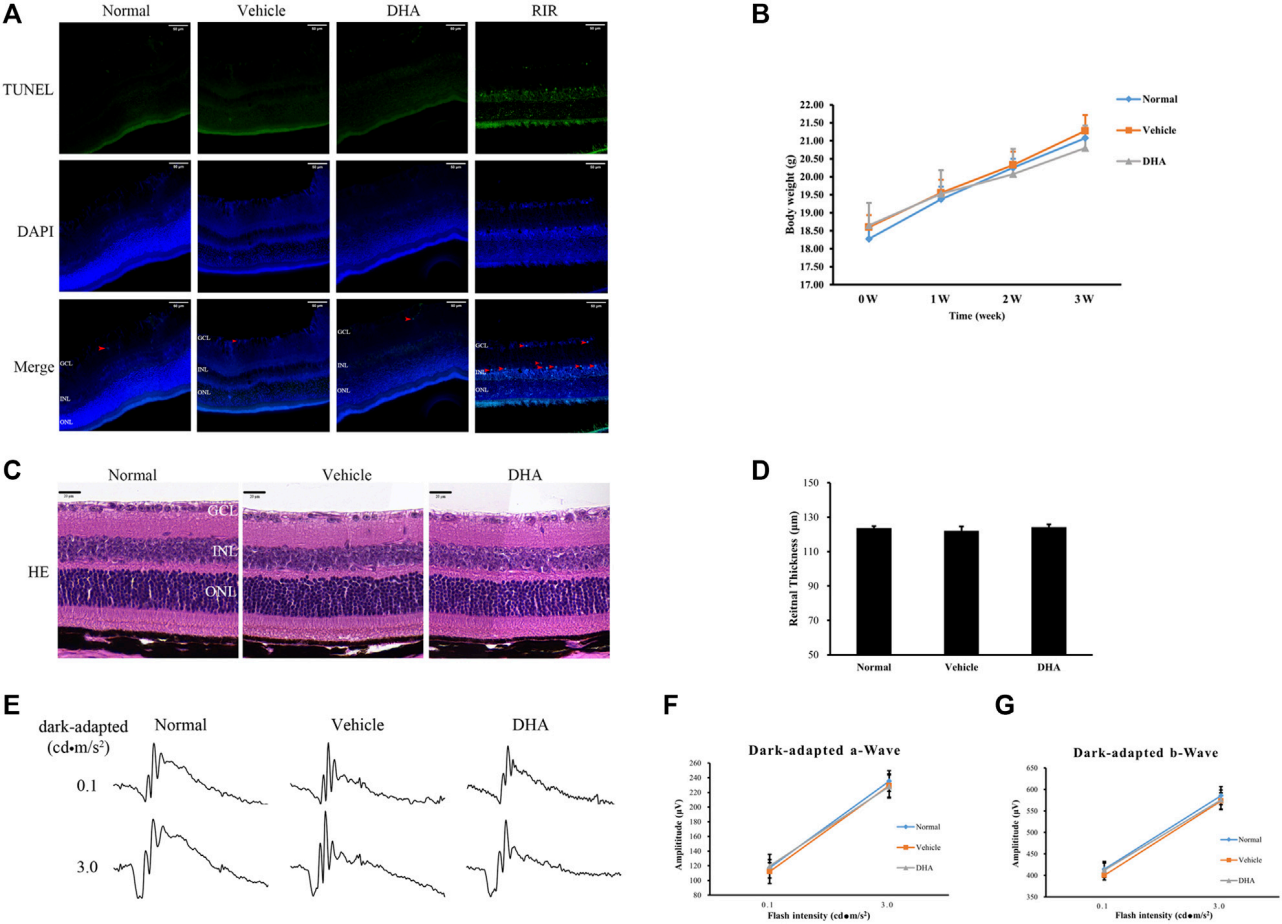
DHA causes no obvious retinal toxicity. **(A)** DAPI (blue) and TUNEL (green) labeling of retinas obtained from the normal, vehicle, and DHA groups. TUNEL + cells were indicated by red arrowheads. The mouse retina at 24 h after ischemia-reperfusion (IOP raised to 110 mmHg for 1 h) was selected as a positive control group (RIR). Compared to the normal group, there were almost no or very few TUNEL + cells in the mice administered vehicle or DHA, and the retinal cell apoptosis was unchanged among these three groups. Scale bar, 50 μm. **(B)** Changes in body weight as a marker for toxicity. **(C)** HE staining of retinal RPE-choroid complex paraffin sections. There were no obvious morphological abnormalities in the vehicle or DHA group compared with the normal group. Scale bar, 20 μm. **(D)** Quantification of the total retinal thickness was shown. **(E)** Representative ERG responses in the normal, vehicle, and DHA groups. **(F)** The ERG amplitude versus flash intensity for dark-adapted a-wave. **(G)** The ERG amplitude versus flash intensity for dark-adapted b-wave. There was no significant difference among these three groups. n = 4/group. RIR, Retinal Ischemia-reperfusion; GCL, Ganglion Cell Layer; INL, Inner Nuclear Layer; ONL, Outer Nuclear Layer; HE, Hematoxylin Eosin; DAPI, 4′,6-diamidino-2-phenylindol; DHA, Dihydroartemisinin; TUNEL, TdT-mediated dUTP nick-end labeling; IOP, Intraocular Pressure.

To confirm the safety of intragastric DHA application, retinal cell death in mice was assessed by TUNEL assay after 3 weeks of administration of DHA or vehicle (Figure 6A). Compared to the normal group, there were almost no or very few TUNEL + cells in the mice administered vehicle or DHA, and the retinal cell apoptosis was unchanged among these three groups. Retinas with ischemia-reperfusion injury (RIR) were selected as a positive control, showing a significant rise in the number of TUNEL + cells in the retina (Figure 6A). All the mice remained alert and responsive during the study periods, and no adverse effects were observed based on body weight (Figure 6B). Similar to the normal mice, the layers of the neural retina in the vehicle or DHA group were closed organized, and the cells were neatly arranged, as shown by HE staining of ocular sections. There were no obvious differences both in histologic morphology (Figure 6C) and retinal thickness (Figure 6D) among the three groups. In addition, the ERG amplitudes versus flash intensity for dark-adapted a-wave and b-wave were recorded and indicated that there was no significant difference among these three groups. These results suggested that intragastric administration of DHA causes no obvious intraocular toxicity within the dosage range used in this study.

## Discussion

**FIGURE 7:**
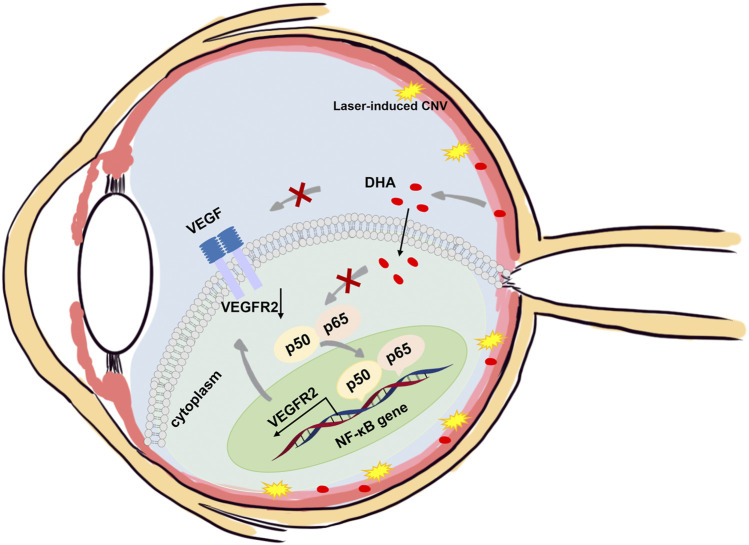
Schematic illustration of the mechanisms of the anti-CNV effects of DHA. In addition to the downregulation of VEGF expression, DHA inhibits VEGFR2 expression and angiogenesis through suppression of the NF-κB pathway.

The present study investigated the efficacy of DHA on experimental laser-induced CNV in mice and showed that intragastric administration of DHA markedly suppressed laser-induced CNV. The activation of NF-κB signaling was related to inflammatory and angiogenesis reactions, which have a key role in the development of CNV, and these changes were notably reduced following DHA administration. These data suggested that DHA might have inhibitory effects on CNV by suppressing NF-κB signaling *in vivo* (Figure 7).

DHA, derived from the Chinese herbal medicine artemisinin, is known as a traditional antimalarial drug and shows anti-inflammatory and antiangiogenic properties (D’Alessandro et al., 2007). The anti-inflammatory effects of DHA have been studied broadly. In a lipopolysaccharide-induced acute kidney injury mouse model, treatment with DHA improved renal function by inhibiting NF-κB-mediated inflammation, ameliorating tubular cell apoptosis, and suppressing oxidative stress (Huang et al., 2019). In endothelial cells, DHA not only reduced prostaglandin (PG) E2 levels but also blunted the upregulation of the inflammatory cytokines interleukin (IL)-6 and IL-1β induced by PGE2, suggesting the potential of DHA for the treatment of inflammatory vascular diseases (Yin et al., 2018). In addition, artemisinin and its derivatives have been reported to be highly effective against a wide variety of tumors (Efferth, 2017). The anticancer activity of DHA partially depends on its antiangiogenic effect, since targeting angiogenesis has become a major strategy for tumor therapy (Dai et al., 2021). Several previous studies demonstrated that DHA can inhibit angiogenesis by reducing the expression of HIFα, VEGF, and VEGF receptors (Huang et al., 2010; Dong et al., 2014; Cheong et al., 2020), which are therapeutic targets of CNV. In a rat model of corneal neovascularization, both 20 mg/L and 10 mg/L DHA could significantly reduce the proportion of NV area to the whole cornea by topical administration (Zhong et al., 2011). Considering the importance of inflammation and angiogenesis in CNV formation, we investigated the role of DHA in a mouse model of laser-induced CNV.

Although the pathogenesis underlying CNV has not been fully elucidated, increasing experimental and clinical studies have revealed that inflammation and NF-κB signaling are critical factors that promote CNV formation. The NF-κB pathway has long been considered one of the potent prototypical proinflammatory signaling pathways, and its role in several aspects of human health has been established (Gambhir et al., 2015). In both colorectal and lung carcinoma, NF-κB plays a key role in pathological angiogenesis, and strategies to inhibit NF-κB are effective in decreasing tumors (Debusk et al., 2008; Sakamoto et al., 2009; Panahi et al., 2016). Izumi-Nagai et al. showed that carotenoids inhibited the activation of NF-κB signaling, including IκB-α degradation and p65 nuclear translocation, leading to the significant suppression of CNV(Izumi-Nagai et al., 2007; Izumi-Nagai et al., 2008). Additionally, the VEGF/VEGFR2 signaling axis drives tumor vascularization by activating proangiogenic signaling in endothelial cells (ECs), which is commonly targeted by antiangiogenic therapies. VEGFR2 has been identified as a downstream target gene regulated by NF-κB signaling that directly binds the VEGFR2 upstream promoter and activates its transcription (Rönicke et al., 1996; Dong et al., 2014). In the present study, we observed that DHA notably suppressed CNV leakage and decreased CNV areas. The protein levels of VEGF, VGFER2, NF-κB p65, and NF-κB1 p50 increased significantly in the eyes of experimental laser-induced CNV, and these changes were profoundly inhibited following DHA administration. These results demonstrated that DHA may inhibit CNV formation by regulating the NF-κB/VEGFR2 signaling axis *in vivo*.

Considering the limitations of anti-VEGF in patients with neovascular AMD, DHA has its own advantages. As one of the most common causes of blindness, neovascular AMD has been a global health problem in the elderly population. Currently, intravitreal anti-VEGF drugs, including ranibizumab, bevacizumab, aflibercept, and brolucizumab, have revolutionized the treatment of neovascular AMD (Mettu et al., 2020). However, many patients suffer from an incomplete response to anti-VEGF therapy, and intravitreal injection causes potential complications, such as vitreous hemorrhage, infection, and retinal detachment. Repeated injections lead to a higher chance of drug resistance and poor patient compliance (Wei et al., 2018). Compared to anti-VEGF therapy, DHA may avoid these disadvantages. First, DHA, used for treating malaria clinically for many years, has been proven to be safe and has fewer side effects *in vivo* (Gutman et al., 2017). DHA within the dosage range in this study caused no obvious abnormalities in detailed retinal morphology and apoptosis of retinal cells, which was analyzed by HE staining and TUNEL assay. Second, DHA can be administered via the oral route, which is more acceptable to patients and prevents the adverse effects caused by intravitreal injection (Okada et al., 2020). Third, as a cost-effective drug, DHA has potential application prospects for the treatment of CNV to reduce the economic burden in patients with neovascular AMD. These advantages give DHA potent applications for the treatment of CNV.

To better evaluate the clinical significance of DHA, only lesions with clinical meaning of CNV were chosen for experiments in this study, which were confirmed by FFA 4 days after laser photocoagulation (Figure 1). Previous studies have shown that experimental CNV can be clearly identified by FFA on day 4 after laser photocoagulation in all cases by leakage and as fibrovascular and tubular structures in histological HE-stained sections (Hoerster et al., 2012). Then, the CNV-inhibiting effect of DHA was compared with intravitreal injections of ranibizumab. Whether in CNV areas or thicknesses, there was no significant difference between the DHA group and ranibizumab group (*p* > .05). These results showed broad clinical application prospects of DHA in the treatment of CNV. However, some questions remained to be addressed in our study. As for the laser-induced CNV mouse model, diminished tubular structures observed by fluorescence and confocal microscopy of perfused flat-mounts showed spontaneous regression of CNV after 2 weeks. This tendency to spontaneous RPE recovery and CNV regression constitutes a major limitation of this model for interventional studies. Furthermore, the potential mechanism by which DHA inhibits CNV may not be limited to the NF-κB/VEGFR2 signaling axis and needs to be elucidated in more detail in future experiments.

In summary, the present study has demonstrated for the first time that systemic administration of DHA attenuates vascular leakage and the formation of CNV through a laser-induced CNV mouse model, which has been used for most neovascular AMD study experiments. This effect might be due to the suppression of the classic NF-κB signaling pathway and downregulation of VEGFR2 and VEGF expression. Our data indicate that DHA could be a natural potential alternative therapeutic strategy for neovascular AMD.

## Data Availability

The raw data supporting the conclusion of this article will be made available by the authors, without undue reservation.
